# Supplementary material for: Multilayer modelling of the human transcriptome and biological mechanisms of complex diseases and traits
Source: NPJ Syst Biol Appl. 2021 May 27;7:24. doi: 10.1038/s41540-021-00186-6 (PMC8160250; doi:10.1038/s41540-021-00186-6)
Supplement: Supplementary file 1 — Supplementary Material [file 41540_2021_186_MOESM1_ESM.pdf]

Supplementary Material
Multilayer modelling of the human transcriptome and biological mechanisms of complex diseases and traits

Tiago Azevedo[1,†], Giovanna Maria Dimitri[1,2,3,†], Pietro Lio[1,2,*], Eric R. Gamazon[2,4,5,6,*]

[1]Department of Computer Science and Technology, University of Cambridge, Cambridge, UK
[2]Clare Hall, University of Cambridge, Cambridge, UK
[3]Department of Engineering, University of Siena, Italy
[4]Vanderbilt Genetics Institute, Vanderbilt University Medical Center, Nashville, TN, USA
[5]Data Science Institute, Vanderbilt University, Nashville, TN, USA
[6]MRC Epidemiology Unit, University of Cambridge, Cambridge, UK
[†]These authors contributed equally to this work.
[*]Correspondences should be addressed to: pl219@cam.ac.uk, ericgamazon@gmail.com.

Methods

Prediction of Tissues Given Reactome Pathways. For comparison with the communities, we investigated the extent to which biologically meaningful sets of genes encoding current biological knowledge are predictive of tissues. For each Reactome [3] pathway, we selected the expression of member genes from the master matrix M. If a gene from a Reactome pathway was not present, that gene was ignored. Using the same stratified 3-fold cross validation procedure described in Methods of the main text, we performed 49 binary classifications.

Embedding new transcriptome data into UMAP learned space. To evaluate the relevance of the trained model generated from the GTEx communities, we passed previously unseen data D_{test} to the model for embedding into the learned latent map (from the UMAP embedding of GTEx training data, $\phi : D_{train} \subset M_g \hookrightarrow \mathbb{R}^m$). We used The Cancer Genome Atlas (TCGA) gene expression data in 33 cancer types.

Results

Relationship between Reactome Pathways and Tissues. Consistent with our observations for the communities, 197 Reactome pathways are not sufficient to predict any tissue (available on github: output *output_06_02*), while 164 are tissue-specific (i.e., can predict only one tissue). However, we identified Reactome pathways that can predict more than half of the tissues: *GPCR LIGAND BINDING*, *GPCR DOWNSTREAM SIGNALING*, and *SIGNALING BY GPCR* predict 34, 33, and 32 tissues, respectively. This observation is perhaps expected: G-protein-coupled receptors (GPCRs) comprise a large family of cell surface receptors that form the essential sites of communication between the internal and external environments of cells, with a central and widespread role in human physiology [4]. Their gene expression profile in each of the predicted tissues differs from the remaining tissues, potentially reflecting their broad but tissue-specific function.

Prediction of tissues by Reactome pathways varies substantially. The brain tissues "Brain Caudate" (basal ganglia), "Brain Frontal Cortex", "Brain Hippocampus", and "Brain Nucleus" accumbens (basal ganglia) are not predicted by any Reactome pathway, likely reflecting the fact that our current understandings (as encoded in these pathways) have been hampered by the relative inaccessibility of these tissues. In contrast, the tissues cells cultured fibroblasts and whole blood are the tissues most highly predicted (269 and 330 Reactomes, respectively). Some tissues are predicted by less than 5 Reactome pathways, including the tissues "Brain Amygdala" (two), "Brain Anterior Cingulate" cortex (BA24) (one), "Brain Cortex" (two), and "Brain Hypothalamus" (two). More information on the relationship between communities and enriched pathways is available on github: notebook *10_reactomes_per_tissue*.

Embedding new transcriptome data into UMAP learned space. Interestingly, embedding of each of the TCGA datasets into the learned latent space from the communities showed clus-

tering with the testis tissue. This result recapitulates two known results: (a) the GTEx finding that the testis is an outlier relative to the other GTEx tissues in transcriptome profile [2] and (b) the role of the so-called cancer-testis (CT) genes [1], which function as driver genes in cancer [6, 7], evoke immune responses in cancer patients as immunogenic antigens across a range of cancers [5], and contribute to various neoplastic phenotypes. The implementation is available on github: notebook *12_tcga*.

TWAS performance gain. To demonstrate the generalisability of the TWAS performance gain observed for the communities, we analysed two additional GWAS datasets in the UK Biobank, haemoglobin concentration (n = 350,474; Supplementary Figure 8) and white blood cell (leukocyte) count (n = 350,470; Supplementary Figure 9), using whole blood gene expression models. We leveraged the GWAS summary statistics (Neale lab; `http://www.nealelab.is/uk-biobank`) for these traits.

References

[1] Y. Chang, X. Wang, Y. Xu, L. Yang, Q. Qian, S. Ju, Y. Chen, S. Chen, N. Qin, Z. Ma, et al. Comprehensive characterization of cancer-testis genes in testicular germ cell tumor. *Cancer medicine*, 8(7):3511–3519, 2019.

[2] G. Consortium et al. The genotype-tissue expression (gtex) pilot analysis: multitissue gene regulation in humans. *Science*, 348(6235):648–660, 2015.

[3] A. Fabregat, S. Jupe, L. Matthews, K. Sidiropoulos, M. Gillespie, P. Garapati, R. Haw, B. Jassal, F. Korninger, B. May, M. Milacic, C. D. Roca, K. Rothfels, C. Sevilla, V. Shamovsky, S. Shorser, T. Varusai, G. Viteri, J. Weiser, G. Wu, L. Stein, H. Herm-

jakob, and P. D'Eustachio. The reactome pathway knowledgebase. *Nucleic Acids Research*, 46(D1):D649–D655, Nov. 2017.

[4] D. M. Rosenbaum, S. G. Rasmussen, and B. K. Kobilka. The structure and function of g-protein-coupled receptors. *Nature*, 459(7245):356–363, 2009.

[5] M. J. Scanlan, A. Simpson, L. J. Old, et al. The cancer/testis genes: review, standardization, and commentary. *Cancer Immun*, 4(1):1, 2004.

[6] A. J. Simpson, O. L. Caballero, A. Jungbluth, Y.-T. Chen, and L. J. Old. Cancer/testis antigens, gametogenesis and cancer. *Nature Reviews Cancer*, 5(8):615–625, 2005.

[7] C. Wang, Y. Gu, K. Zhang, K. Xie, M. Zhu, N. Dai, Y. Jiang, X. Guo, M. Liu, J. Dai, et al. Systematic identification of genes with a cancer-testis expression pattern in 19 cancer types. *Nature communications*, 7(1):1–12, 2016.

Supplementary Tables

Supplementary Table 1: **Information about communities in each tissue.** Statistics (i.e., mean, std, median, and MAD) refer to the distribution of community size for each tissue

Tissue	Number of genes without a community	Number of communi-ties with size below 4	Total number of communi-ties	Mean	Std	Median	MAD
Adipose Subcutaneous	15770	69	84	3.167	2.6763	2.0	0.0
Adipose Visceral Omentum	15730	62	91	4.055	4.2462	2.0	0.0
Adrenal Gland	15260	93	125	4.648	7.0373	2.0	0.0
Artery Aorta	15463	73	100	4.1	4.2837	2.0	0.0
Artery Coronary	15490	80	112	5.286	8.2163	2.0	0.0
Artery Tibial	15402	58	78	3.718	3.9545	2.0	0.0
Brain Amygdala	15430	71	102	7.255	13.5536	2.0	0.0
Brain Anterior cingulate	15499	67	96	6.938	11.868	2.0	0.0
Brain Caudate	15748	57	89	5.36	7.5032	2.0	0.0
Brain Cerebellar	15402	106	136	5.029	8.0229	2.0	0.0
Brain Cerebellum	15713	88	113	4.327	5.2131	2.0	0.0
Brain Cortex	15765	60	91	5.747	8.6642	2.0	0.0
Brain Frontal Cortex	15638	58	89	6.798	9.861	2.0	0.0
Brain Hippocampus	15642	67	103	5.981	10.0242	2.0	0.0
Brain Hypothalamus	15885	66	94	6.223	10.6553	2.0	0.0
Brain Nucleus	15725	63	86	5.709	8.3525	2.0	0.0
Brain Putamen	15470	70	96	5.604	9.0559	2.0	0.0
Brain Spinal cord	15550	93	129	6.24	12.7788	2.0	0.0
Brain Substantia nigra	15241	99	139	6.669	11.8099	2.0	0.0
Breast Mammary Tissue	16050	77	98	3.704	4.7385	2.0	0.0
Cells Cultured	14525	75	94	3.723	6.1995	2.0	0.0
Cells EBV	14052	151	185	3.784	5.7673	2.0	0.0
Colon Sigmoid	15811	70	92	4.391	6.0666	2.0	0.0
Colon Transverse	15946	83	116	4.06	4.1028	2.0	0.0
Esophagus Gastro	15609	60	86	4.64	5.8387	2.0	0.0
Esophagus Mucosa	15556	77	105	4.038	4.4953	2.0	0.0
Esophagus Muscularis	15573	64	86	4.14	5.0513	2.0	0.0
Heart Atrial	15265	51	76	4.908	5.9564	2.5	0.5

Continued on next page

Supplementary Table 1: **Information about communities in each tissue.** Statistics (i.e., mean, std, median, and MAD) refer to the distribution of community size for each tissue

Tissue	Number of genes without a community	Number of communi-ties with size below 4	Total number of communi-ties	Mean	Std	Median	MAD
Heart L Vent	14654	51	76	4.276	4.6043	2.0	0.0
Kidney Cortex	14667	192	251	6.116	14.1071	2.0	0.0
Liver	14769	80	116	5.026	8.0456	2.0	0.0
Lung	16090	74	99	4.152	4.5911	2.0	0.0
Minor Salivary	15545	97	137	6.124	11.8639	2.0	0.0
Muscle Skeletal	14746	63	73	2.863	2.4231	2.0	0.0
Nerve Tibial	15975	66	84	3.798	4.228	2.0	0.0
Ovary	15478	109	142	4.62	8.6917	2.0	0.0
Pancreas	15297	59	80	4.012	5.0855	2.0	0.0
Pituitary	16318	81	108	4.102	5.2651	2.0	0.0
Prostate	16149	87	115	4.287	6.2147	2.0	0.0
Skin Not Sun Epsd	16023	74	99	3.939	4.559	2.0	0.0
Skin Sun Epsd	16082	64	87	3.598	3.12	2.0	0.0
Small Intestine	15680	101	145	6.172	12.3027	2.0	0.0
Spleen	15455	83	111	5.342	9.4654	2.0	0.0
Stomach	15767	67	96	4.906	6.9463	2.0	0.0
Testis	17679	70	91	3.538	3.3946	2.0	0.0
Thyroid	15994	93	111	3.216	2.8582	2.0	0.0
Uterus	15473	113	148	4.649	8.0395	2.0	0.0
Vagina	15596	99	132	6.227	13.1912	2.0	0.0
Whole Blood	14061	69	85	3.447	3.5695	2.0	0.0

Supplementary Table 2: **Sample size of each tissue in GTEx V8.**

Tissue	Sample Size
Adipose Subcutaneous	581
Adipose Visceral Omentum	469
Adrenal Gland	233
Artery Aorta	387
Artery Coronary	213
Artery Tibial	584
Brain Amygdala	129
Brain Anterior cingulate	147
Brain Caudate	194
Brain Cerebellar	175
Brain Cerebellum	209
Brain Cortex	205

Continued on next page

Supplementary Table 2: **Sample size of each tissue in GTEx V8.**

Tissue	Sample Size
Brain Frontal Cortex	175
Brain Hippocampus	165
Brain Hypothalamus	170
Brain Nucleus	202
Brain Putamen	170
Brain Spinal cord	126
Brain Substantia nigra	114
Breast Mammary Tissue	396
Cells Cultured	483
Cells EBV	147
Colon Sigmoid	318
Colon Transverse	368
Esophagus Gastro	330
Esophagus Mucosa	497
Esophagus Muscularis	465
Heart Atrial	372
Heart L Vent	386
Kidney Cortex	73
Liver	208
Lung	515
Minor Salivary	144
Muscle Skeletal	706
Nerve Tibial	532
Ovary	167
Pancreas	305
Pituitary	237
Prostate	221
Skin Not Sun Epsd	517
Skin Sun Epsd	605
Small Intestine	174
Spleen	227
Stomach	324
Testis	322
Thyroid	574
Uterus	129
Vagina	141
Whole Blood	670

Supplementary Figures

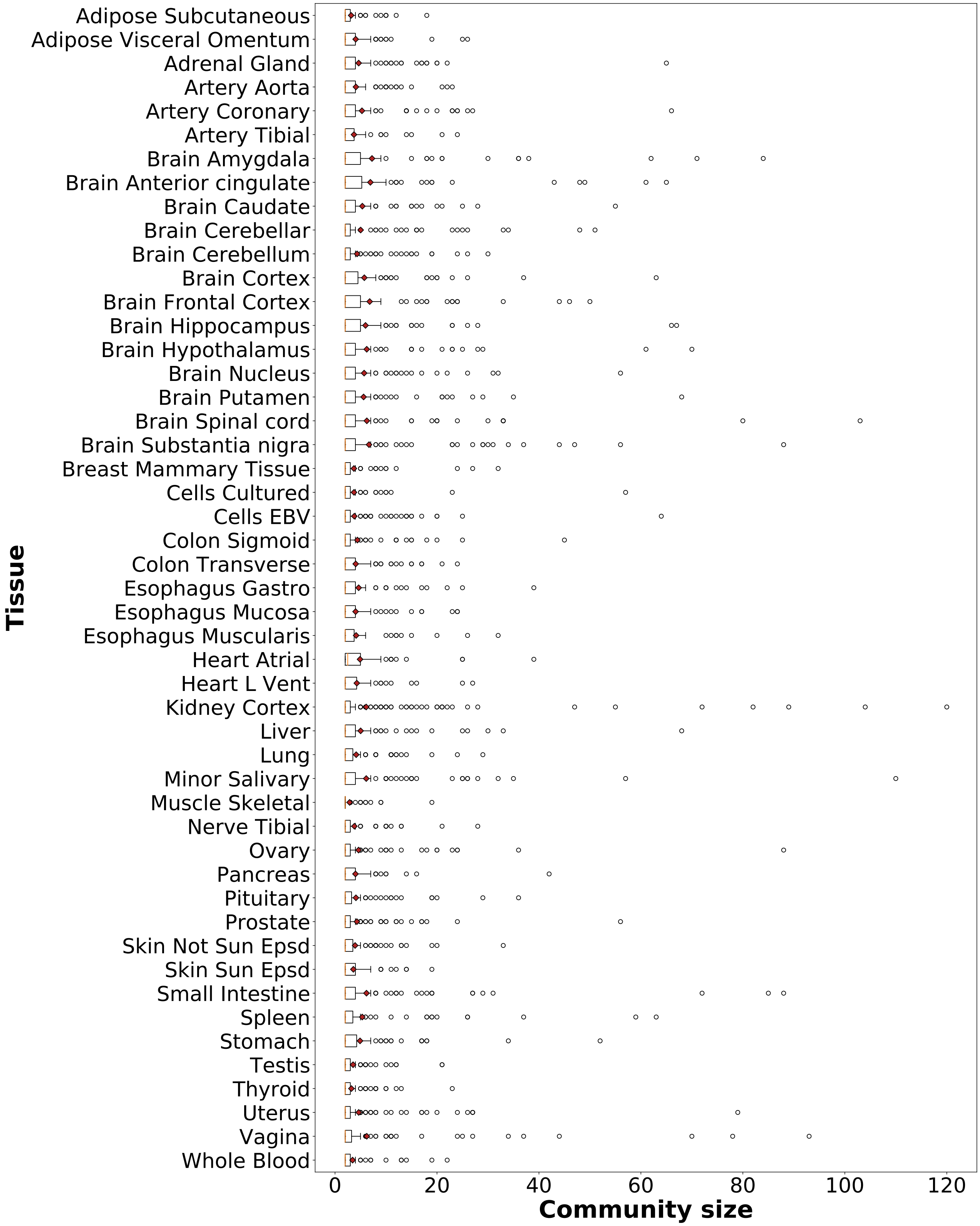

Supplementary Figure 1: **Distribution of community size for each tissue.** The boxplots show the distribution of community size for each of the 49 tissues. Community size varies within each tissue and across tissues.

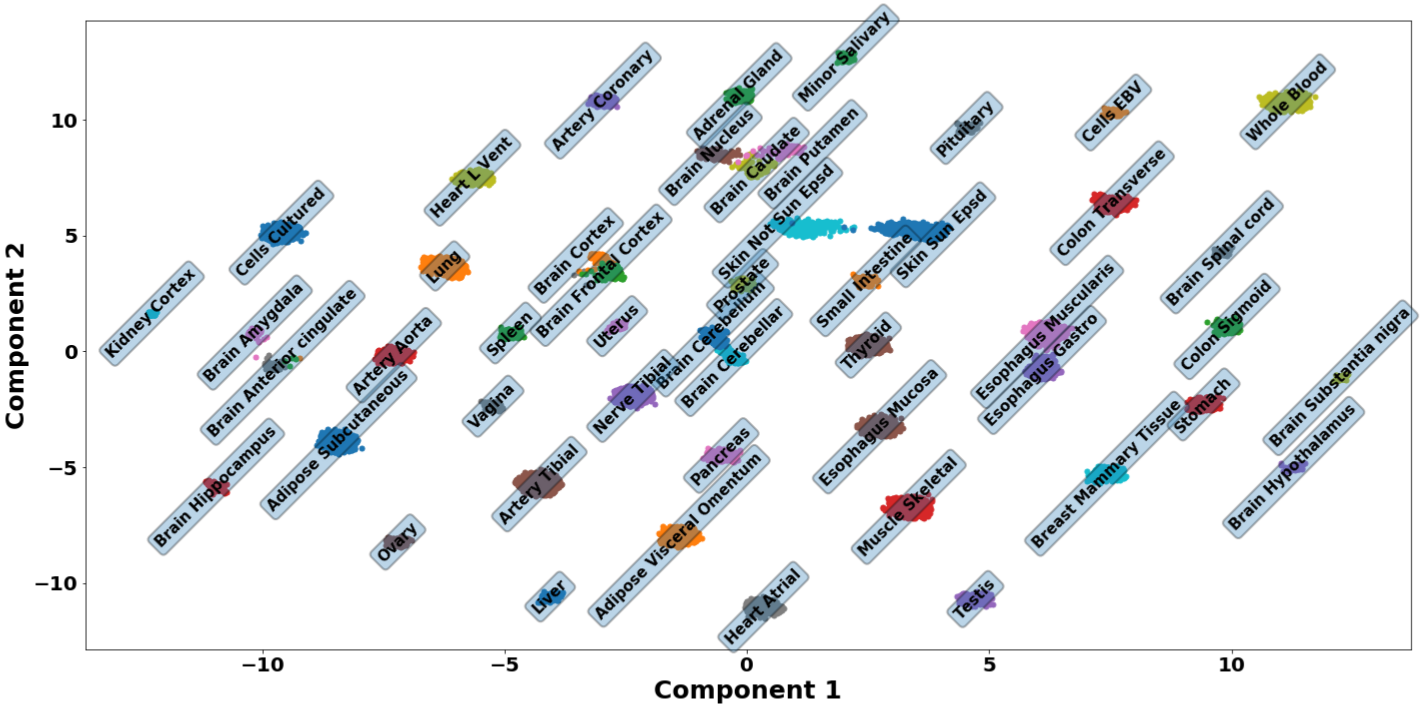

Supplementary Figure 2: **Lower-dimensional representation of the GTEx transcriptome data.** The plot shows the UMAP embedded components from the full GTEx master matrix consisting of all genes. The clusters formed in the embedding space are well-defined. The UMAP parameters used to create the reduced embeddings assumed the default values except for the size of local neighbourhood (i.e., 5), the effective minimum distance between embedded points (i.e., 0.25), the learning rate (i.e., 0.5), and the spread (i.e., 1).

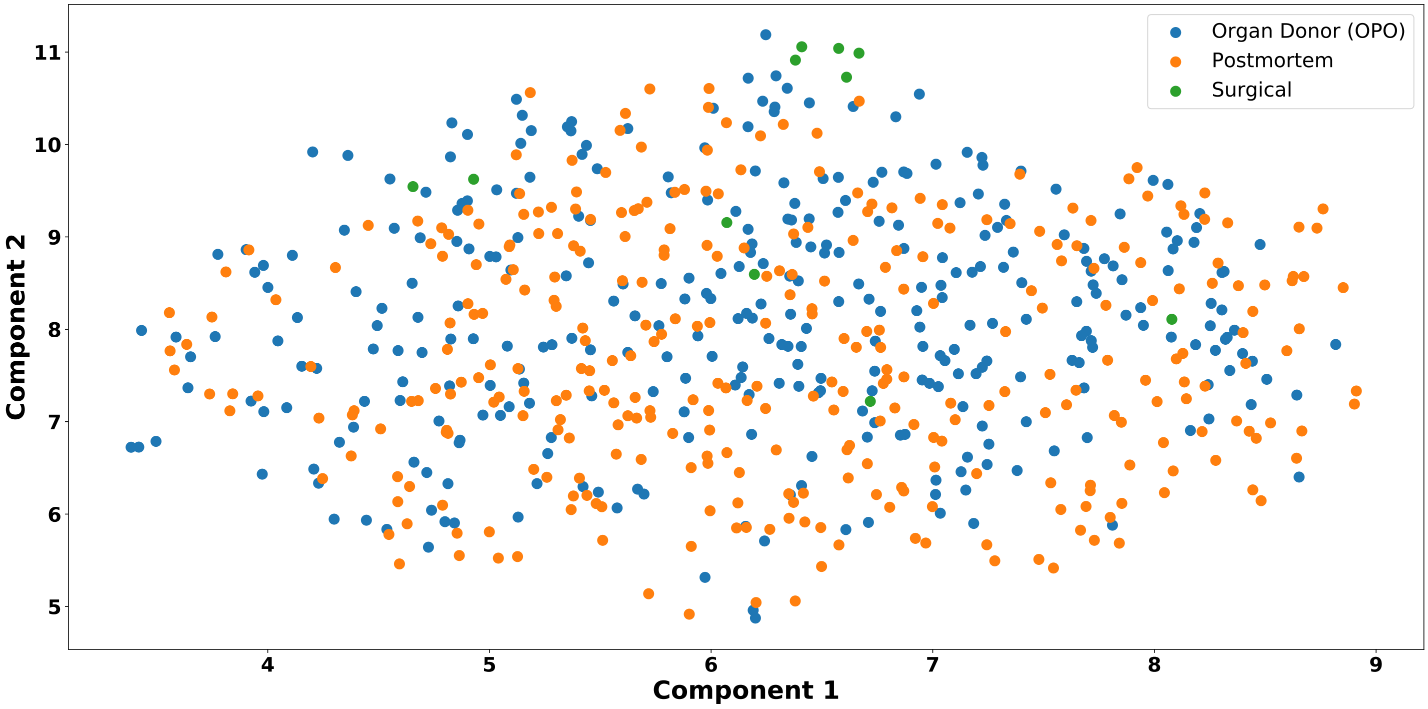

Supplementary Figure 3: **Lower-dimensional representation of the whole blood transcriptome, overlayed with COHORT values.** The UMAP embedding shows no clustering in the whole blood structure with regards to COHORT, indicating that this confound was successfully corrected with *sva*.

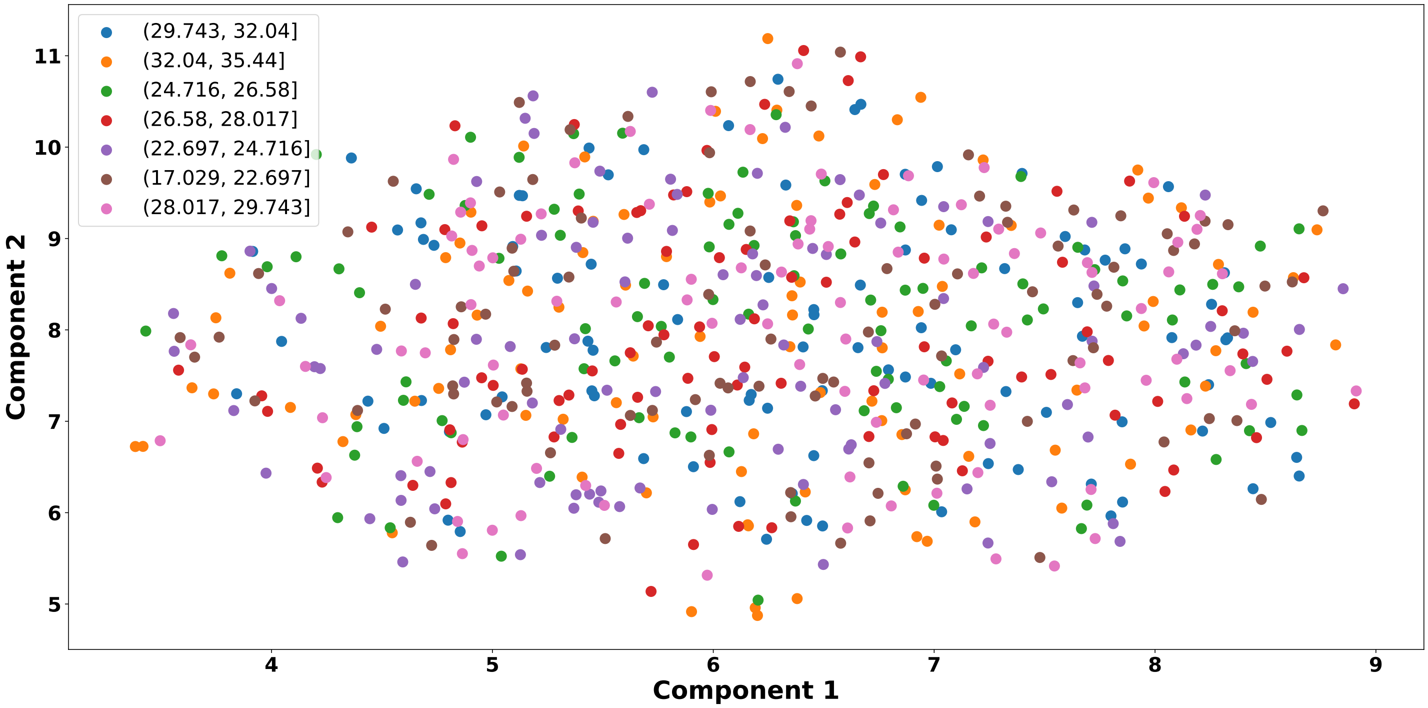

Supplementary Figure 4: **Lower-dimensional representation of the whole blood transcriptome, overlayed with BMI values.** The UMAP embedding shows no clustering in the whole blood structure with regards to BMI, indicating that this confound was successfully corrected with *sva*.

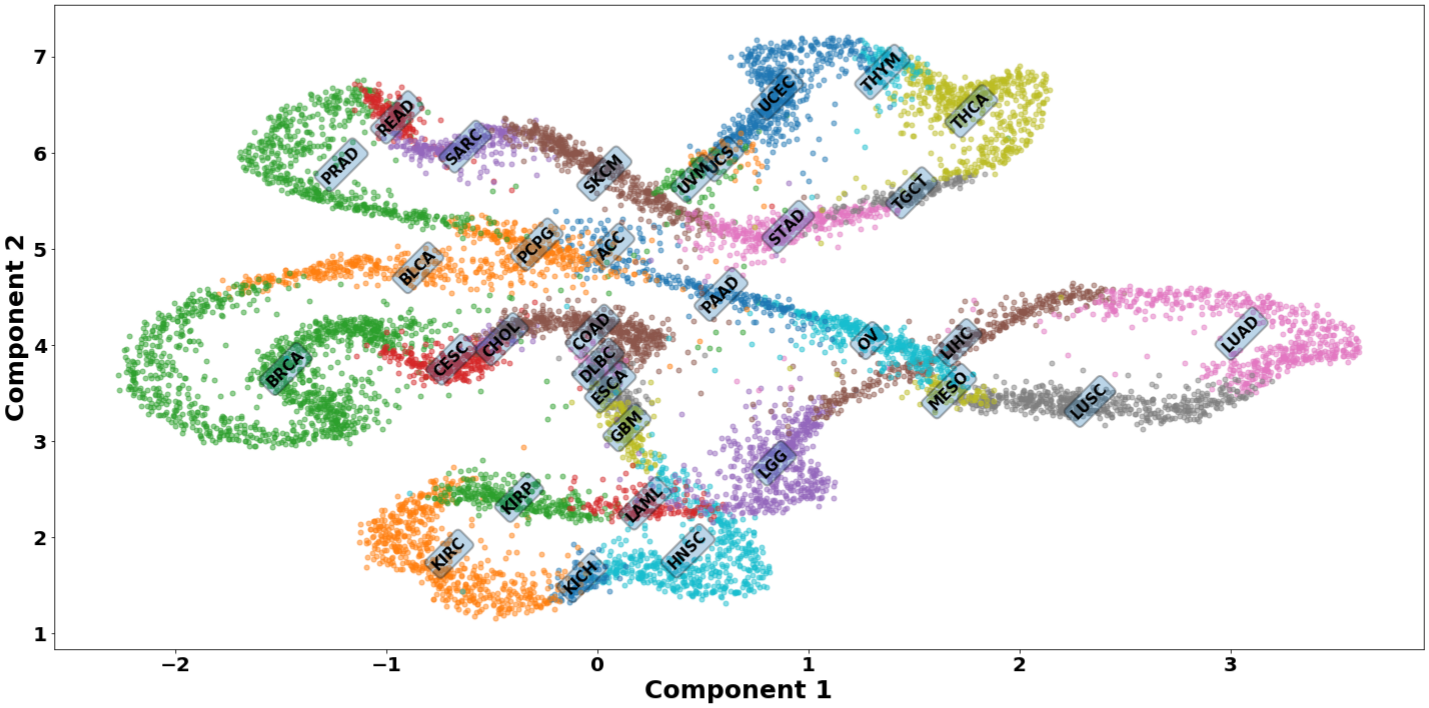

Supplementary Figure 5: **Lower-dimensional representation of the GTEx-derived communities in TCGA transcriptome data.** The plot shows the UMAP embedded components through a low-dimensional projection of the submatrix consisting of only the genes that belong to a community in at least one GTEx tissue ($n = 3,259$). The UMAP parameters used to create the reduced embeddings assumed the default values except for the size of local neighbourhood (i.e., 5), the effective minimum distance between embedded points (i.e., 0.25), the learning rate (i.e., 0.5), and the spread (i.e., 0.3).

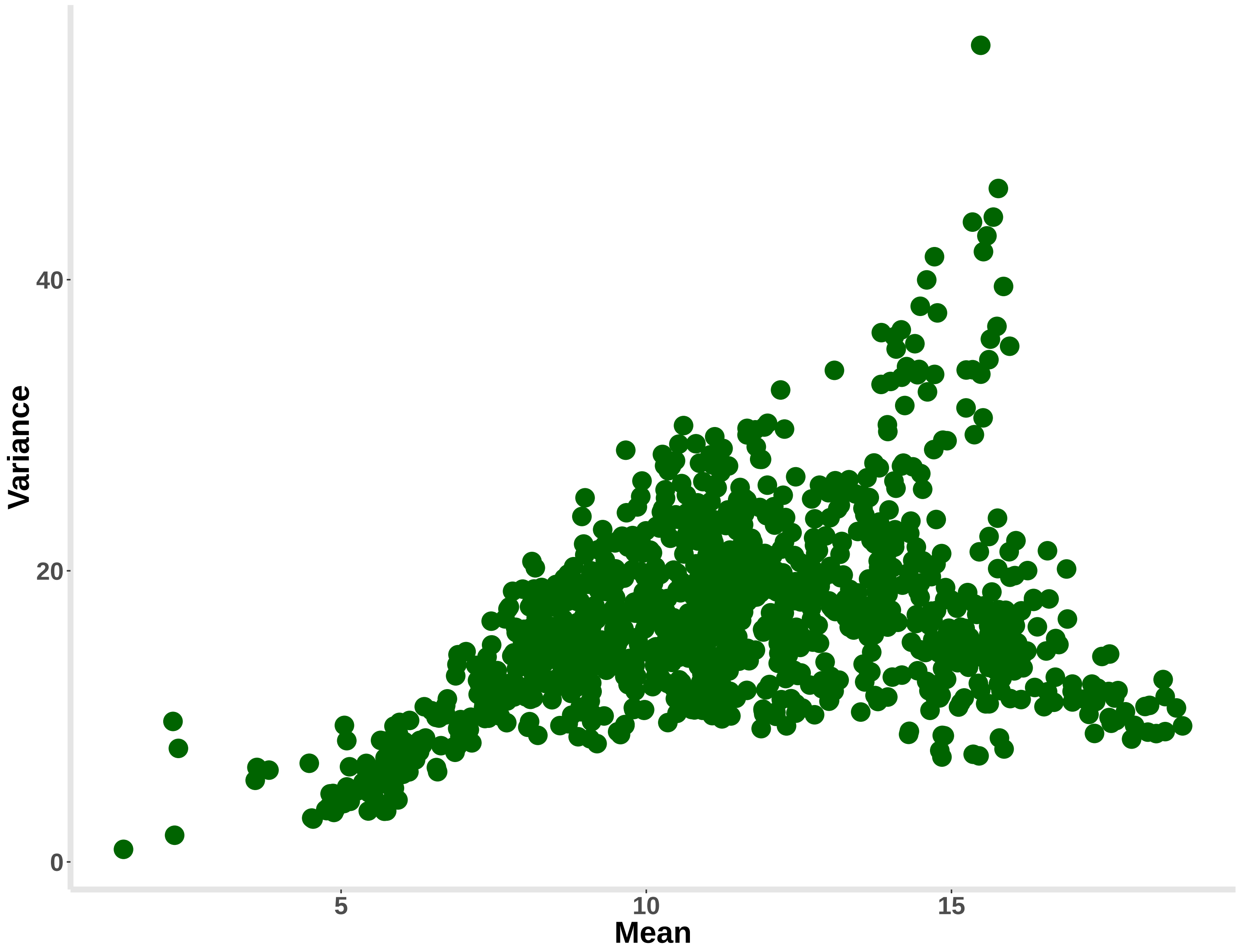

Supplementary Figure 6: **Mean and variance of distance between clusters across bootstrapped manifolds.** There is a significant correlation between mean and variance (Spearman $\rho \approx 0.38$, $p < 2.2 \times 10^{-16}$) across bootstraps. For a pair of tissue clusters, greater average distance implies greater variability in the distance.

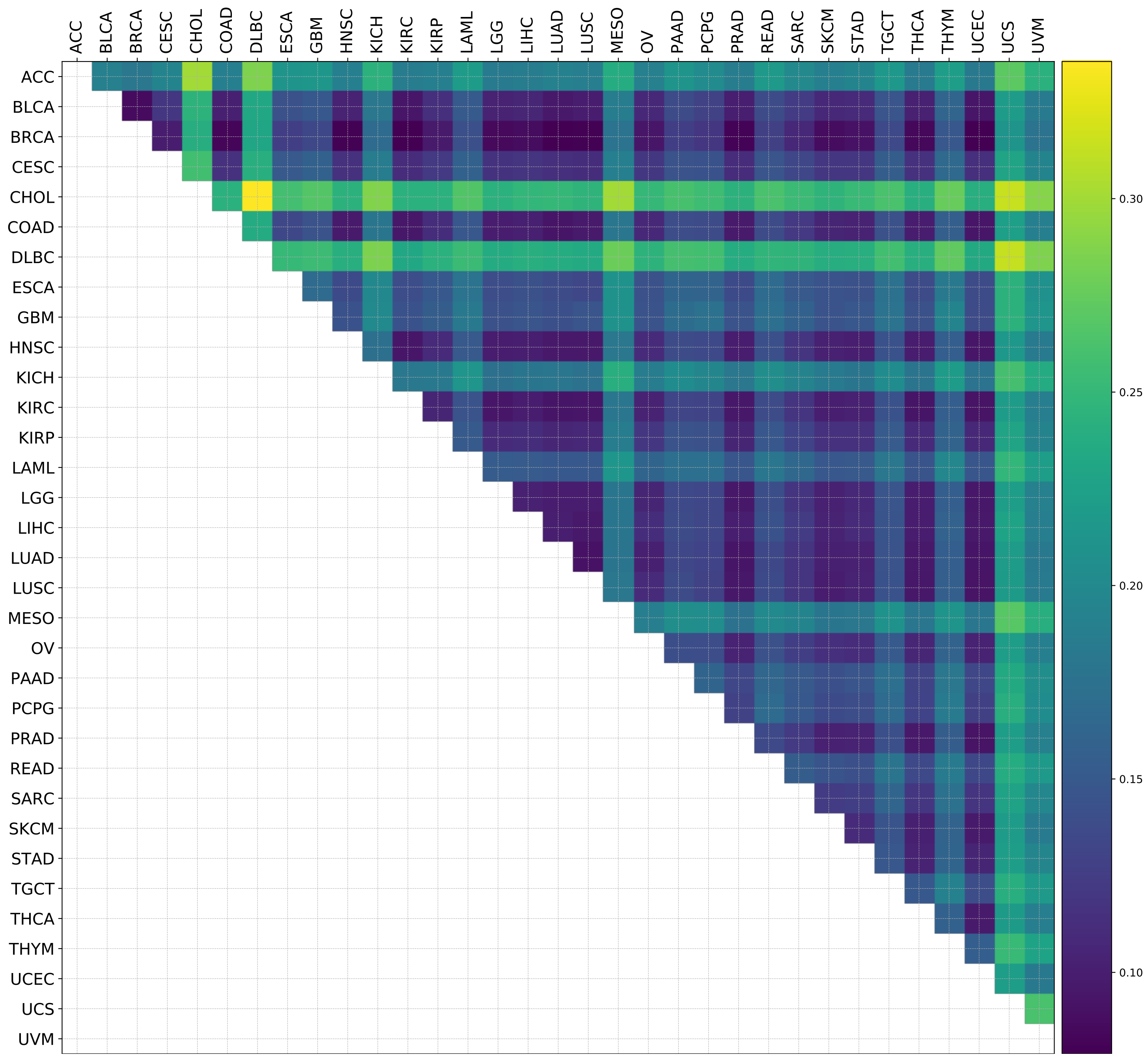

Supplementary Figure 7: **Conservation of global structure on the lower-dimensional representation of GTEx-derived communities in TCGA transcriptome data.** Using bootstrapped manifolds (see Methods), we estimated the persistence of the global structure and pairwise relationships across TCGA cancer types. Here we show the upper-triangular matrix of the average pairwise distances across the bootstrapped manifolds.

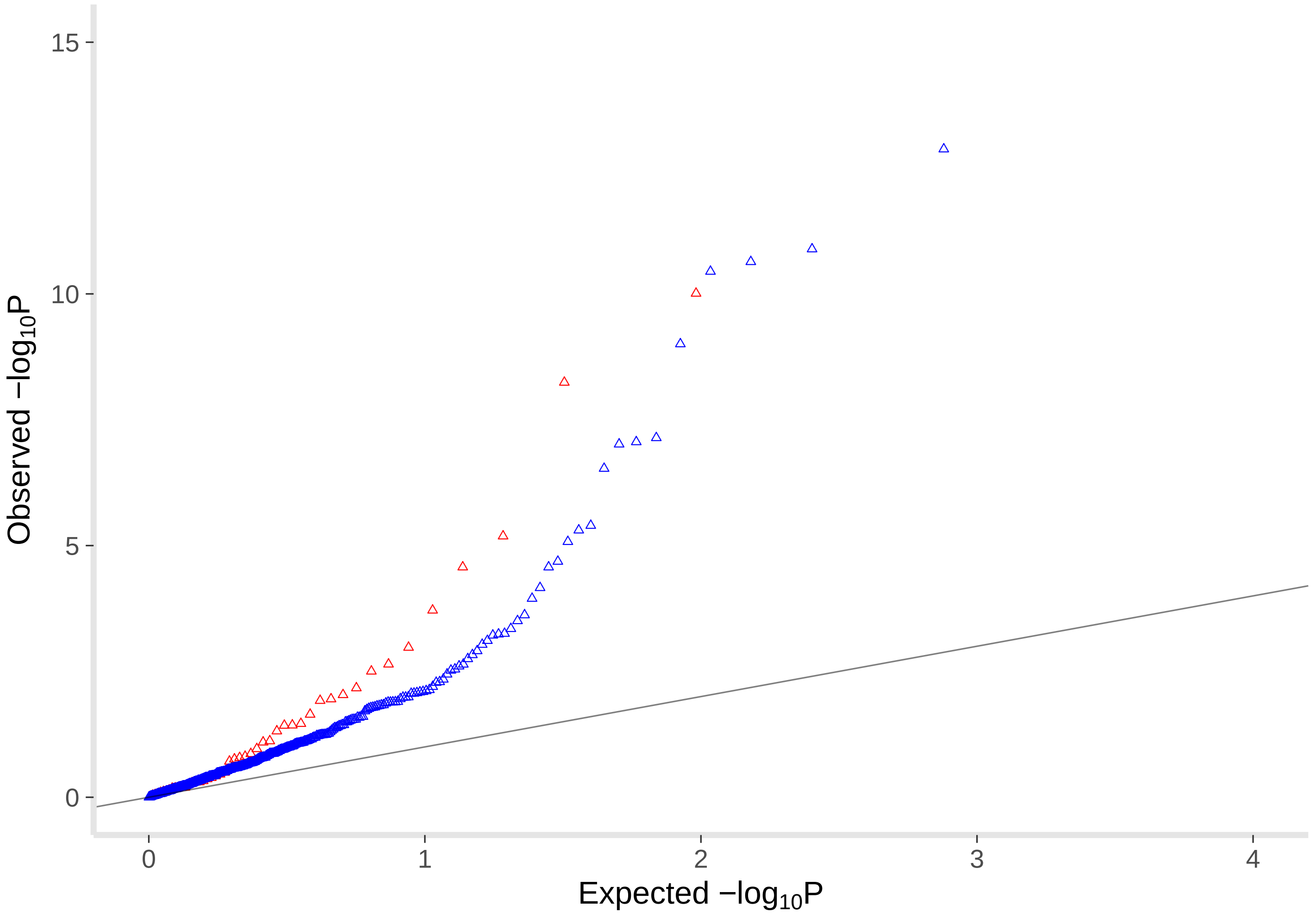

Supplementary Figure 8: **TWAS performance gain in identifying genes associated with haemoglobin concentration.** The set of genes in the communities (red) displayed a greater departure from the null expectation (i.e., greater enrichment for significant associations) than the complement set of genes (blue), as shown by the leftward shift in the Q-Q plot.

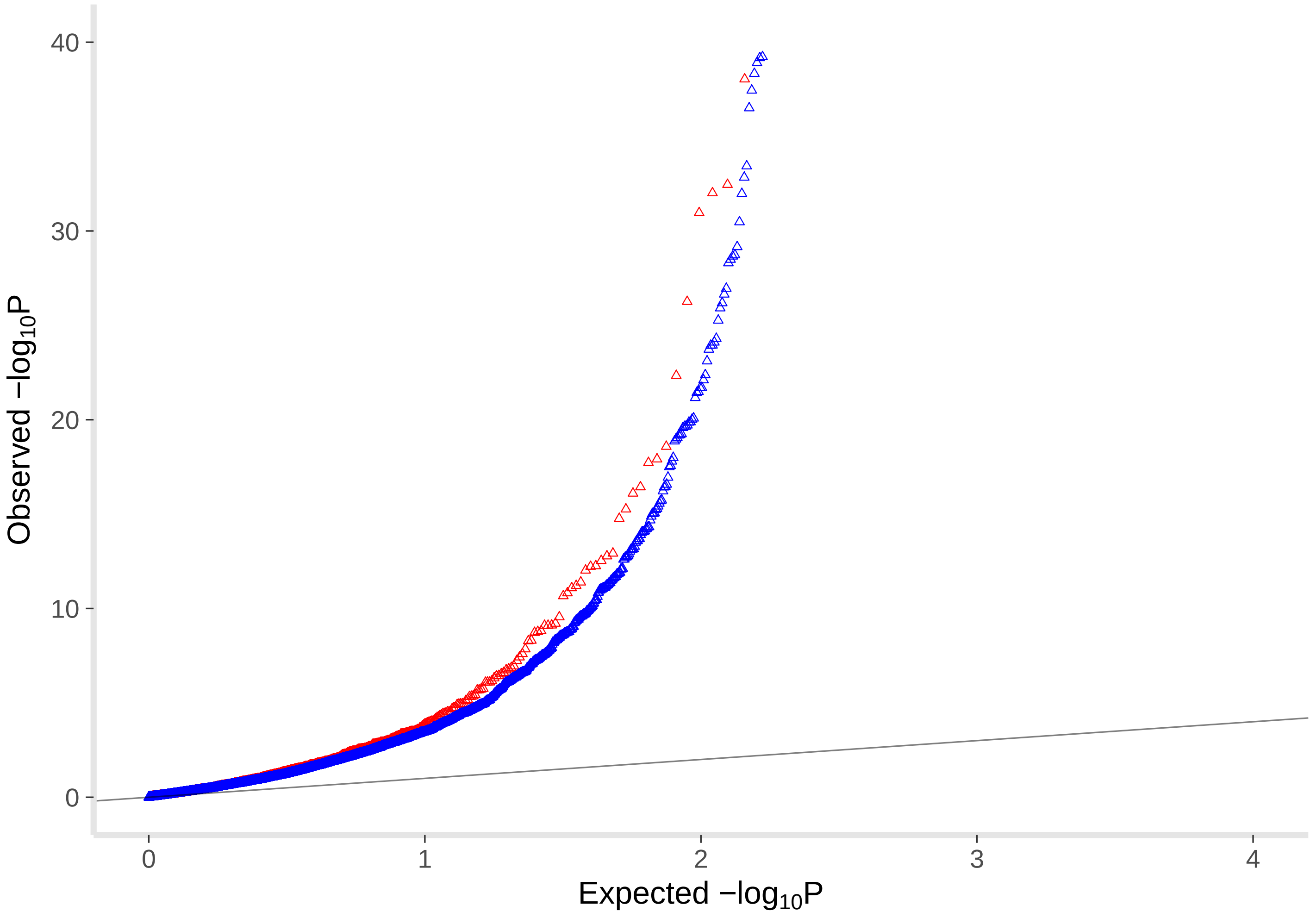

Supplementary Figure 9: **TWAS performance gain in identifying genes associated with white blood cell (leukocyte) count.** The set of genes in the communities (red) displayed a greater departure from the null expectation (i.e., greater enrichment for significant associations) than the complement set of genes (blue), as shown by the leftward shift in the Q-Q plot.

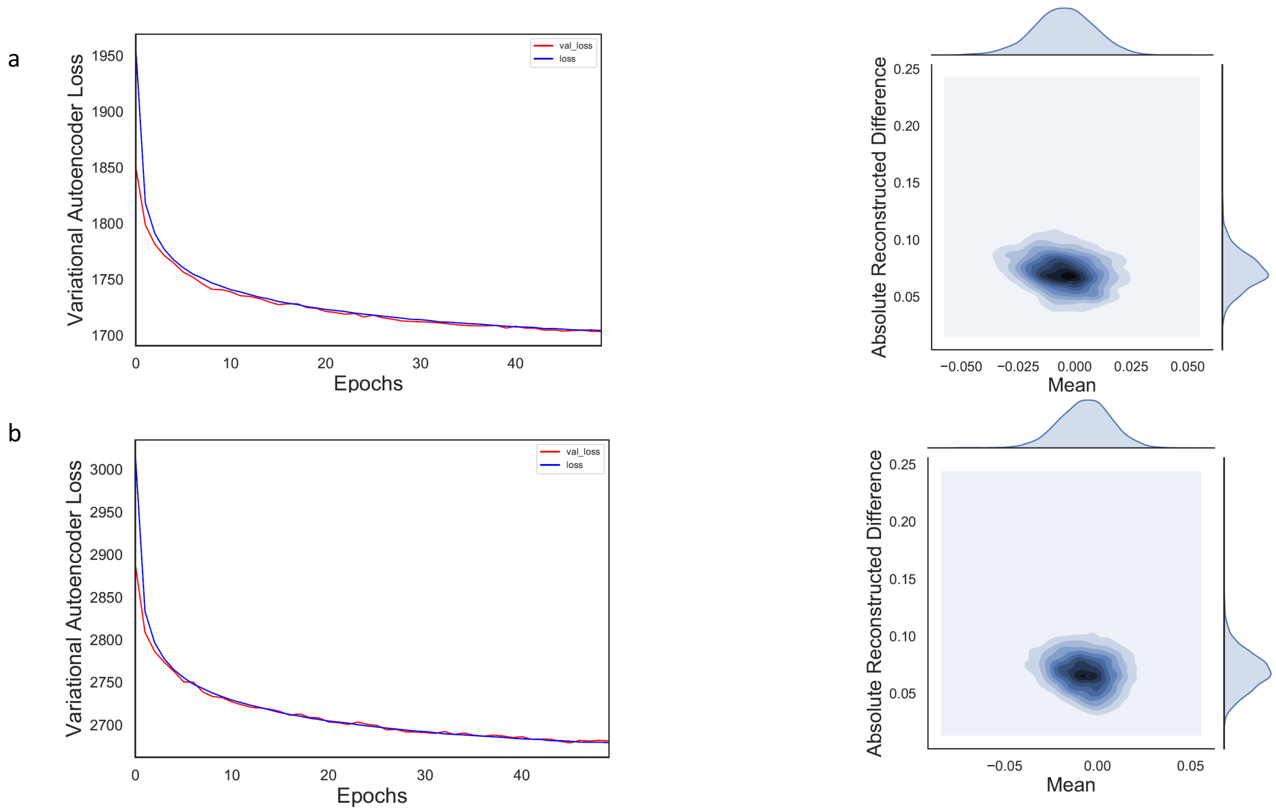

Supplementary Figure 10: **VAE diagnostics.** **a.** The training performance (VAE loss as a function of epoch number) and reconstruction accuracy (mean vs absolute difference) of the VAE applied to the genes in the communities are shown (top panel). **b.** The corresponding plots for the full set of genes regardless of community membership are shown (bottom panel). *Tybalt* code was modified to generate these diagnostic plots.
